# Advancing the Conservation and Utilization of Barley Genetic Resources: Insights into Germplasm Management and Breeding for Sustainable Agriculture

**DOI:** 10.3390/plants12183186

**Published:** 2023-09-06

**Authors:** Andrea Visioni, Boris Basile, Ahmed Amri, Miguel Sanchez-Garcia, Giandomenico Corrado

**Affiliations:** 1 International Center for Agricultural Research in the Dry Areas (ICARDA), Rabat 10100, Morocco; a.amri@cgiar.org (A.A.); m.sanchez-garcia@cgiar.org (M.S.-G.); 2 Department of Agricultural Sciences, University of Naples Federico II, 80055 Portici, Italy; boris.basile@unina.it

**Keywords:** *Hordeum vulgare*, genetic diversity, crop improvement, biodiversity, climate resilience, landraces, participatory breeding approaches, genetic resource centers

## Abstract

Barley is a very important crop particularly in marginal dry areas, where it often serves as the most viable option for farmers. Additionally, barley carries great significance in the Western world, serving not only as a fundamental crop for animal feed and malting but also as a nutritious food source. The broad adaptability of barley and its ability to withstand various biotic and abiotic stresses often make this species the sole cereal that can be cultivated in arid regions. The collection and utilization of barley genetic resources are crucial for identifying valuable traits to enhance productivity and mitigate the adverse effects of climate change. This review aims to provide an overview of the management and exploitation of barley genetic resources. Furthermore, the review explores the relationship between gene banks and participatory breeding, offering insights into the diversity and utilization of barley genetic resources through some examples such as the initiatives undertaken by ICARDA. Finally, this contribution highlights the importance of these resources for boosting barley productivity, addressing climate change impacts, and meeting the growing food demands in a rapidly changing agriculture. The understanding and utilizing the rich genetic diversity of barley can contribute to sustainable agriculture and ensure the success of this vital crop for future generations globally.

## 1. Introduction

### 1.1. The Diversity and Main Uses of Barley: An Outline of the Plant Domestication, Adaptation, Agronomic Classifications, and Health Claims

Barley (*Hordeum vulgare* L.) is one of the most adaptable crops belonging to the Poaceae family, serving as a vital source of food, feed, and malt for the brewing industry on a global scale [1]. This self-fertile species is characterized by a diploid genome (2n = 2x = 14), although tetraploid (2n = 4x = 28) and hexaploid (2n = 6x = 42) species are also described in *Hordeum*. A high-quality genome sequence was achieved following the establishment of the International Barley Genome Sequencing Consortium (IBSC) in 2006. This endeavor was based on two main approaches: the BAC-by-BAC strategy, which exploited a large set of genetically mapped BAC clones, and whole-genome shotgun sequencing, synergized with deep RNA-Seq analysis [2,3]. A physical map encompassing 4.98 Gbp of the haploid barley genome size (5.10 Gbp) has been successfully constructed, with a substantial portion (exceeding 3.90 Gbp) aligned and anchored to a high-resolution genetic map [2]. The genome was found to be rich in repeat sequences (more than 80% of the total genome) [2]. Later, chromosome conformation capture sequencing (Hi-C) was employed to order and orient super-scaffolds. The final chromosome-scale assembly represents about 95% of the barley genome [3]. At extra-nuclear level, it was found that chloroplast sequences from cultivated and wild barley were closely related [4,5]. The mitochondrial genomes of wild and cultivated barley were also compared, showing only minimal differences in terms of sequence and structure [6]. As expected, fragmented organelle sequences are reported in the barley nuclear genome [3,6].

Barley has been cultivated and selectively bred for thousands of years, resulting in abundant genetic and morphological diversity [7,8]. Although there are significant taxonomic disagreements, particularly for the delimitations within the various species of the *Hordeum* genus, all the cultivated forms are classified as *H. vulgare* subsp. *vulgare*, with the wild progenitor considered conspecific (*H. vulgare* subsp. *spontaneum* (K. Koch) Asch. et Graebn.); hereinafter *H. spontaneum*). This classification is supported by phylogenetic analysis and the ability of these plants to interbreed [9]. A key morphological distinction between wild and cultivated barley is the characteristic of the rachis (i.e., the main axis of the spike). Wild barley exhibits a brittle rachis and when it reaches maturity, the spike releases its triplets as dispersal units. On the other hand, cultivated varieties possess a tough rachis that retain the intact spike, providing a fundamental trait for domestication.

Among cultivated forms, various types are commonly distinguished. These distinctions are often based on characteristics such as growth habit, row type (two-rowed or six-rowed), and the presence/absence of hulls [10,11]. All the species of the genus have three one-flowered spikelets, with one central spikelet and two lateral spikelets arranged alternately at each rachis node. In two-rowed and wild barley, the lateral spikelets are smaller in size and are sterile, while in six-rowed barley cultivars, all three spikelets are fertile and capable of developing into grains. The two-rowed spikes of the wild barley provide an evolutionary advantage in nature, favoring seed dispersal and germination. The presence of a six-rowed spike was determined by a single allele called *vrs1* (previously known as *v* for *vulgare*), which is recessive to the dominant allele responsible for the two-rowed spike (*Vrs1*). Throughout domestication, the *Vrs1* gene has undergone independent mutations multiple times, resulting in the emergence of the six-rowed phenotype [12]. This is accompanied by the presence of *vrs5*, an allele that enhances lateral grain fill characteristics to modern six-row cultivars [13]. Recent studies have unveiled the molecular basis of five major row-type genes (*Vrs1* to *Vrs5*), all involved in the regulation of the arrangement and development of spikelets [14].

Coming to the different types of barley, hulled barley is encased in a tough outer layer, while hulless (naked) barley, scientifically known as *Hordeum vulgare* L. var. *nudum* Hook. f., lacks this outer layer. In relation to adaptability, winter barley is more suitable to colder climates and typically sown in the fall, while spring barley thrives in warmer climates and is usually sown in the spring in higher latitudes and in fall in the Mediterranean climate. Furthermore, variants can be classified based on agronomic characteristics. Finally, certain types of barley are particularly suitable for malting due to their content of amylase and proteins. Protein content in barley seeds ranges from 8% to 30% of the total seed mass [15,16,17,18]. Generally, for malting total protein, it should be from 9 to 12%, while alpha amylase activity should be higher than 150 U/g [19]. These varieties are widely used in the production of malt for the brewing industry, syrups, and other non-alcoholic beverages. Other types of barley are better suited for food production (e.g., baked goods, soups, stews, breakfast cereals, but also couscous, bulgur, and other types of mixed grain-based products commonly used in various cuisines), animal feed (primarily for livestock such as cattle, pigs, and poultry), and various biotechnological applications (e.g., enzyme purification, cosmetics, pharmaceuticals) [1,10,11]. 

Recently, barley has gained renewed attention as a food source particularly with the advancement of biofortified varieties that are enriched with beta glucans [20], a type of soluble fiber found also in oat (*Avena sativa*) and some other cereals. Beta glucans are known for their ability to lower cholesterol levels, regulate blood sugar levels, and promote digestive health. They have been linked to a reduced risk of heart disease, improved immune function, and enhanced weight management [21,22]. By incorporating biofortified barley into common food products (e.g., bread, biscuits, cereal breakfast), it would be possible to provide consumers with an affordable and enjoyable way to integrate the health benefits of beta glucans into their daily diet. A scientific opinion on beta-glucans has been provided by the European Food Safety Authority (EFSA) [23]. The claim pertaining to maintaining normal blood LDL-cholesterol concentrations was assessed with a favorable outcome, while the claim regarding an increase in satiety, which should lead to a reduction in energy intake, is not supported by a confirmed cause-and-effect relationship between the consumption of beta-glucans and sustained satiety. The claim for the reduction of post-prandial glycemic responses was confirmed by a cause-and-effect relationship, with a recommended wording “Consumption of beta-glucans from oats or barley contributes to the reduction of glucose rise after a meal”. It is recommended to consume 4 g of beta-glucans from oats or barley for every 30 g of available carbohydrates per meal to achieve this effect. Finally, the claim regarding an improvement in digestive function was deemed insufficiently defined because it lacks clarity (i.e., the claim does not specify the targeted nutrients) [23]. 

### 1.2. Aim of the Review

**Figure 1:**
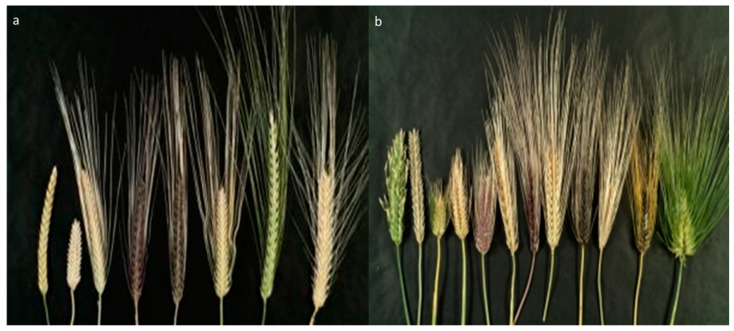
Morphological variations in barley head types: (**a**) examples of two-rowed and (**b**) examples of six-rowed ears.

Barley possesses extensive phenotypic and genetic diversity, due to its long history of cultivation and adaptation to diverse environments worldwide, which is preserved in governmental or international gene banks and germplasm collections in research institutions, universities, breeding companies, and agricultural organizations across the globe (Figure 1).

The progress made in genomics and biotechnology has facilitated the identification and characterization of genes and molecular markers associated with essential agronomic traits in barley [24]. These advancements have not only led to the development of new barley varieties but have also deepened our understanding of the diversity and utility of barley genetic resources [25,26]. For example, exome sequencing and a combination of genome-wide analyses of a set of barley geographically diverse landraces revealed significant associations between days to heading and plant height with seasonal temperature and dryness variables, suggesting that these traits were major drivers of environmental adaptation in the sampled germplasm. A further detailed analysis of flowering time genes showed that patterns of single and multiple haplotypes exhibit strong geographical structure that have contributed to the wide ecogeographical adaptation of barley [27]. 

This review aims to provide an assessment of the genetic resources of barley, including its wild relatives and cultivated varieties. It will explore the management and exploitation of these genetic resources, highlighting the importance of strategies such as gene bank establishment and core collections’ development. The review will also delve into the management and exploitation of barley genetic resources, considering participatory breeding approaches and the importance and limitations of including local knowledge and perspectives. Finally, the historical contributions of the International Center for Agricultural Research in the Dry Areas (ICARDA) in the exploration and utilization of barley genetic resources will be highlighted. We conclude by discussing how advanced genomics and phenomics tools can be harnessed and exploited by institutions in an equitable manner, prioritizing principles of fairness and equal access among different institutions or organizations globally involved in the conservation of barley genetic resources.

## 2. Genetic Resources of Barley

There is little doubt that environmental changes, agricultural intensification, urbanization, land degradation, and economic factors have significantly contributed to loss of traditional farming practices and, hence, crop diversity in barley, as well as in many other crop species [28,29]. To limit this loss, several political, economic, social, and scientific strategies should be implemented. Among them, the documentation, conservation, and characterization of barley’s genetic resources are central to promote the continued use of barley’s genetic diversity. The achievement of these activities includes a variety of approaches and procedures, which are summarized below.

### 2.1. In Situ Conservation of Barley Genetic Resources: Main Natural Habitats and Traditional Cultivation Areas Worldwide

In situ conservation of genetic resources involves their preservation in their habitats [30]. Specifically, this approach aims at maintaining the genetic diversity of plant populations in their natural environment, but it also requires the protection of traditionally cultivated areas from land degradation and over-exploitation [30]. Barley is conserved on-farm in various regions where it has adapted to specific environmental conditions. These regions are often areas with a long history of cultivation (mainly within traditional farming systems) or are represented by the natural habitats of its wild relatives. 

Briefly, it is possible to identify the following main agro-ecological regions in which barley has thrived and adapted: the Near Eastern area (i.e., Fertile Crescent including Asia Minor and the Caucasus), the European–Siberian area, the Ethiopian area, the East Asiatic area, and New World centers (Americas and Oceania). In each area, the combination of geographic isolation and significant climatic variations has led to the emergence of an ample set of agro-ecological groups of varieties that also include further subdivisions [8].

The Fertile Crescent, which is considered the area where barley was domesticated, is home to a diverse range of wild and cultivated varieties of barley [24]. This region exhibited a vertical zonality and a diverse range of environmental and climatic factors, leading to the development of various barley types, including two-rowed hulled and naked forms, as well as six-rowed barley with different spike densities. Barley is probably the primary cereal crop in regions with less than 300 mm of annual rainfall, where there are significant fluctuations in rainfall in terms of amounts and distribution. In the Middle East and North Africa, barley is mainly grown as animal feed. The two-row barley is possibly the most common form of barley currently found in Syria and Turkey, while six-row forms are predominant in Jordan, Tunisia, and Lebanon. Landraces have been described in virtually every area of the Fertile Crescent, such as Syria, Tunisia, Lebanon, Jordan, Iraq, and Turkey [7,31,32,33,34]. In North African countries, six-row landraces remain prevalent, primarily due to limited adoption of improved varieties and the perception of barley as a risk-averse crop among the majority of farmers. These farmers often rely on traditional practices and show minimal or no uptake of certified seeds and other agricultural inputs. 

It is also worth adding the widespread occurrence of wild barley, which can be found across a vast geographic range spanning from Morocco to China [35,36]. Among the 45 taxa of the *Hordeum* genus, wild barley is the sole wild representative in the primary gene pool, and it is the only wild species utilized for genetic improvement in cultivated barley [9,37]. Wild barley (*H. spontaneum*) is normally considered a weed in barley and wheat fields, but it can also be used as forage for livestock. This plant presents a significant amount of genetic variation related to various traits such as biomass, yield, nitrogen content, drought and salinity tolerance, and resistance to diseases [38]. 

The Ethiopian highlands are another important region for in situ conservation, with several barley landraces that are considered important for food security and livelihoods in the region [39]. In Ethiopia, barley is predominantly cultivated as landraces across all regions by subsistence farmers with limited (if any) use of fertilizers, pesticides, or herbicides. Barley is considered a major source of protein in highlands also because of limited alternative crops. The recent introduction of malting varieties has led to a shift among farmers towards cultivating malting barley to boost their incomes, resulting in an overall diversification in the types of barley being grown, with an increasing focus on meeting the demands for both animal feed and human consumption. Ethiopian landraces have been instrumental in the development of powdery mildew-resistant barley varieties in Northwestern Europe. This resistance is conferred by a naturally occurring recessive resistance allele at the *Mlo* gene (mlo-11), most likely present in landraces from the highlands in the southwest region of Ethiopia collected during German expeditions in the 1930s [40]. 

In the East Asiatic area, the Himalaya is another important center of diversity for barley, and the region is home to several wild and cultivated varieties of the crop. Among the former, it is necessary to cite *Hordeum agriocrithon* Åberg, a wild species discovered by Åberg in 1938 known because of its resistance to abiotic and abiotic stress [41]. Among cultivated varieties in Central Asia, noteworthy mentions include the Tibetan, Nepalese, Ladakhi, Bhutanese, Kinnauri, Kumaoni, and Sikkim barley [42,43,44,45,46,47]. These groups of varieties are named after the regions or ethnic groups where they are traditionally grown or have originated from. Although thorough comparative evaluations are not available, each of these barley varieties may have distinct characteristics, including variations in growth habits, yield potential, grain quality, and adaptation to specific environmental and cultural factors such as altitude and culinary traditions (including the production of specific beverages) [42,43,44,45,46,47]. It had been reported that East Asiatic barley is generally characterized by short straw, early maturity, and a high temperature requirement during ripening [26]. 

The Americas and Oceania have a relatively recent history of barley development compared to other regions and are dominated by six-row types. In South America, barley is a valuable staple crop and even today represents an important source of food and income for local communities. Although introduced, this crop is embedded in some traditional cultures, playing a role in traditional festivals and rituals. For example, the Puno region (Peru) is home to the Feast of the Virgen de la Candelaria, a festival that includes drinking of chicha, a traditional fermented beverage made from the two-rowed Andean barley [48]. The Andean region is home to unique varieties of barley because of their adaptation to specific environmental conditions. The two-rowed Andean barley, often classified as *Hordeum vulgare* var. *coeleste*, is well suited to the high altitude and cool temperatures of the region [49]. The six-rowed Andean barley is more common in the lower altitude areas and is often used for animal feed or to produce malt for the brewing industry. The Andean regions are also the origin of *H. chilense* Roem. et Schult., a wild species of barley, known for its crossability with the genera *Triticum* and *Secale* [50,51]. 

### 2.2. Ex Situ Conservation of Barley Genetic Resources: The Role of the Gene Banks

**Figure 2:**
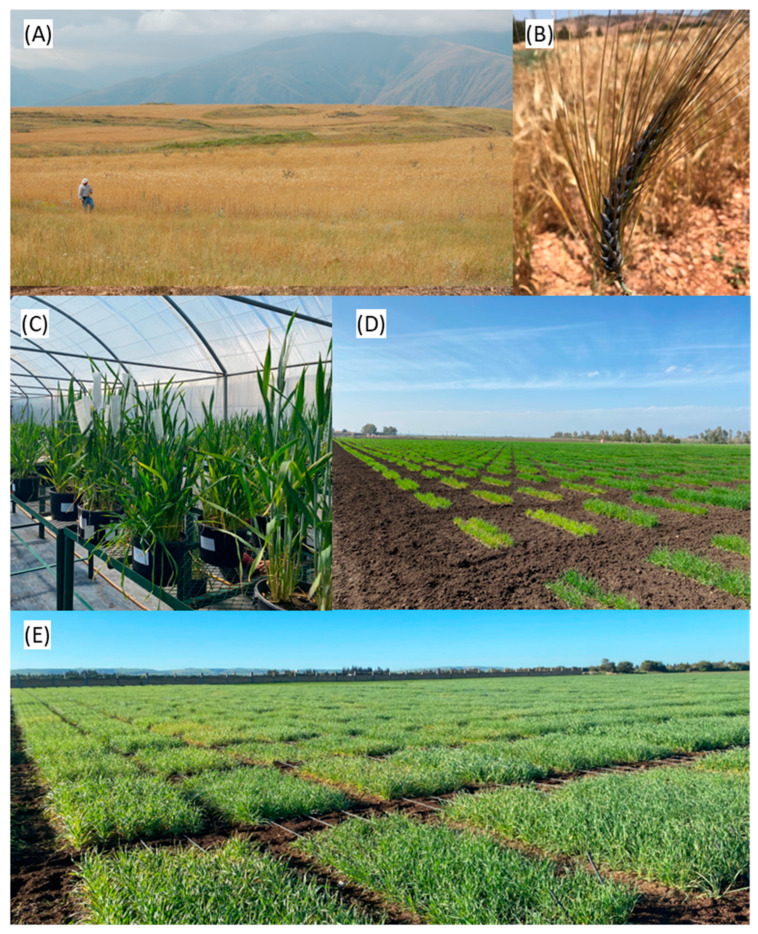
Illustrated workflow for barley germplasm collection and utilization. This figure provides a visual representation of the step-by-step process involved in collecting and utilizing barley germplasm for breeding and research purposes. (**A**) Expedition: Expeditions are organized to collect barley samples from diverse geographical regions, with the highlands of Georgia provided as an example. These expeditions may encompass remote and challenging environments, ensuring a broad range of genetic diversity is captured. (**B**) Collection: Diverse barley accessions, exemplified by the barley with dark-colored grains in the picture, are identified, collected, and documented. This stage also involves carefully cataloging the barley samples for further utilization at experimental stations. (**C**,**D**) Characterization, multiplication, and regeneration: Collected barley accessions are cultivated often under optimum field conditions or less frequently, under greenhouses, and are characterized for major descriptors and agronomic traits. This step also ensures an abundant supply of seeds for distribution to users for evaluation for breeders sought traits. (**E**) Field trial: This panel depicts a field trial specifically focused on drought tolerance. Barley accessions with potential drought tolerance traits undergo rigorous field evaluations under drought-stress conditions. The primary objective of this stage is to identify promising genotypes for further breeding programs and the development of climate-resilient barley varieties.

Ex situ conservation of barley includes establishment of gene banks, botanical gardens, and other facilities that store plant genetic resources. Figure 2 visually reports the key steps and core activities to collect and manage the plant germplasm. Even though different approaches can be used, barley is mainly conserved as seed material, whose longevity is usually highly increased by reducing seed moisture (3–7%) and temperature (around −18 °C) during storage [52,53].

Barley is well-represented in gene banks, evidenced by the considerable number of accessions available. The species is considered among the 14 most important crops for food security by the International Union for Conservation of Nature (IUCN) Red List of Threatened Species, a comprehensive inventory of the conservation status of plant and animal species worldwide. However, more diversity can be collected targeting more wild relative species and populations with adaptive traits to climate change. According to the Second State of the World’s Plant Genetic Resources for Food and Agriculture report published by the Food and Agriculture Organization of the United Nations (FAO) (http://www.fao.org/3/i1500e/i1500e.pdf; accessed on 1 May 2023), there are a total of 466,531 barley accessions (including all taxa) in ex situ collections, and this genus ranks third following *Triticum* and *Oryza*. Nonetheless, as for many other plant species, the level of duplication is not well defined, with estimates indicating that around 120,000 accessions could be distinct (http://www.fao.org/3/i1500e/i1500e.pdf; accessed on 1 August 2023). 

A global inventory of barley genetic resources in ex situ collections was performed through the development of the Global Strategy for the Ex Situ Conservation and Use of Barley Germplasm. This collaborative approach was led by the ICARDA, the Global Crop Diversity Trust, and the Leibniz Institute of Plant Genetics and Crop Plant Research (IPK) (Gatersleben, Germany), with the support of CGIAR and FAO, and the contribution from international barley experts (https://www.genebanks.org/resources/publications/barley-strategy-2008/; accessed on 1 August 2023). The inventory revealed that there are 47 major barley gene banks (holding more than 500 accessions), with a total of 402,000 accessions [11]. The major ones include the following: the Plant Gene Resources of Canada (PGRC), Saskatoon, with around 40,000 accessions, the USDA National Small Grains Collection in Aberdeen (Idaho, USA) and the Recursos Genéticos e Biotecnologia of EMBAPRA (Brazil), the ICARDA-CGIAR, with each having around 30,000 accessions, the John Innes Centre in UK and IPK in Germany, each with holding exceeding 20,000 accessions, and the NordGen’s, with more than 12,000 barley accessions [26,54]. PGRC is a gene bank that maintains a global collection consisting of wild, cultivated varieties and genetic resources for specific traits of interest. Many of the barley accessions are six rowed, although there are also several naked types. The collection includes Canadian and North American varieties, as well as a diverse range of accessions from around the world. The USDA maintains a large gene bank of barley (wild, cultivated, breeding line, etc.) from around the world, with an estimated 18% of national origin. The Recursos Genéticos e Biotecnologia of the Brazilian Agricultural Research Corporation focuses on various aspects of genetic resources and biotechnology, including crop improvement. ICARDA, an international research organization and member of the CGIAR group, has the global mandate for the improvement in barley productivity in dry areas, and maintains a unique and diversity rich collection with more than 32,400 accessions, with approximately two-thirds of landraces [55]. While major descriptors have been used to characterize the ICARDA’s accessions, only a subset of 2400 accessions have been genotyped thus far. NordGen’s collection by a large part (over 11,000) is made of cultivated germplasm, with a focus on locally adapted landraces (mainly to the Nordic and Arctic regions). The collection also includes breeding lines, mutants, and hundreds of wild relatives. The IPK besides a large collection of barley, has developed a deeply phenotyped core collection of 1000 genotypes [56].

## 3. Leveraging Barley Resources: Utilization, Participatory Activities, and Capacity Enhancement in Breeding

### 3.1. Participatory Breeding Approaches to Empower Farmers for Genetic Diversity Conservation and Variety Selection

Participatory approaches involve local communities in the conservation and use of plant genetic resources. Farmers play a central role in identifying, selecting, and preserving local varieties, landraces, and wild relatives, thereby contributing to the overall genetic diversity and adaptive potential of the barley germplasm. Participatory Plant Breeding (PPB) refers to a breeding approach that involves farmers and other stakeholders by actively engaging them in variety cultivation, evaluation, and selection. In barley, PPB has long been an important complementary process within the broader context of preserving and exploiting biodiversity [57]. A key feature is that this approach is expected to ensure that breeding outcomes are context specific and able to address local challenges and needs, including the resistance or resilience to stresses prevalent in specific environments. On the other hand, PPB faces challenges in not being supported by large, centralized seed production and marketing systems. These systems are currently dominated by a small number of corporations not only for barley, but also for a number of arable crops. A main obstacle in integrating the PPB more widely in barley is the requirement for a different seed-managing system that can accommodate the agrobiodiversity fostered by PPB over time and across different locations. Small seed companies should be sustained by a model that goes beyond traditional profit-driven approaches, recognizing the value of farmers’ knowledge and prioritizing long-term benefits. Since 1991, ICARDA has been gradually decentralizing barley selection work to national programs, paving the way for experiments in several countries, including Syria, Egypt, Jordan, Tunisia, Morocco, Yemen, and Eritrea [34]. These programs have generated valuable insights, including the capacity of farmers to handle breeding material and properly select traits of interest, and also confirmed the feasibility and potential of the approach [34]. Briefly, this experience indicated significant differences between the lines selected by breeders on research stations and those chosen by farmers in their fields and a limited phenotypic correlation between research stations and farmers’ fields. Moreover, farmers primarily focused on grain yield as a selection criterion, while also considering other traits including grain filling, straw yield, and the color of straw and leaves, due to the crop’s importance as animal feed [34,58]. Notably, disease resistance received much less emphasis from farmers compared to breeders [34]. 

PPB, despite demonstrating its efficiency, has not been widely embraced mainly because of the reluctance in accepting the “implicit paradigm shift regarding seed sovereignty” [59]. To address this, Evolutionary Participatory Breeding (EPB) was proposed as an alternative. This approach involves planting mixtures of diverse genotypes of the same crop in farmers’ fields, preferably using early segregating generations that should maximize (allelic) diversity, especially for the trait(s) of interest. The creation of a diverse, mixed population, along with the repeated sowing without the active selection of individual genotypes, represents the main conceptual and technical differences within PPB. Over successive crop cycles in the same environment, the genetic composition of the whole population will differ from the originally planted seeds because it is expected that the population will gradually evolve and adapt to the specific environmental conditions such as soil type, fertility, agronomic practices, rainfall, temperature, and more. As climatic conditions differ from year to year, the genetic makeup of the population should be considered always dynamic, but the fraction of genotypes more adapted to that environment will progressively become more prevalent, while the relatively less amenable genotypes will represent a genetic reservoir to guarantee resilience to extreme conditions and long-term adaptation [59]. In essence, EPB retains the main advantages of PPB but is more effective in reintroducing an ample diversity in farmers’ fields without necessarily relying on the long-term support of a scientific institution and formal breeding activities.

The improvement in landraces through participatory selection also encompasses the enhancement of seed quality through seed cleaning and treatment against seed-borne diseases, which have proven to be effective approaches for increasing yields of landraces and contribute to the on-farm conservation of landraces [60].

Irrespective of the approach employed, the active involvement of farmers in the breeding process strongly decreased the risk of developing cultivars that do not meet their preferences or needs. Participatory plant breeding is crucial to preserve germplasm and sustain cultivation for indigenous communities in agroecological niches or marginal lands but also to address needs of specific stakeholders such as smallholder famers, local food processing and brewing industries, consumer groups and associations focused on food quality, safety, and nutrition, and more generally, of agricultural systems that do not rely heavily on off-farm input. To provide an example in cereals, Morocco has experienced a significant increase in wheat productivity through the replacement of local landraces with contemporary cultivars. However, the campaign to replace cultivars halted in the early 1990s, and therefore, most of the currently cultivated varieties were released decades ago. Although breeding programs have continuously released improved varieties, their adoption has been typically low [61], and only recently, we are observing a rising trend towards the adoption of new cultivars, mainly because traditional farming methods are merging with modern agricultural techniques. Coming to barley, it is estimated that landraces still make up over 70% of the cultivated material. Even if different political and socio-economic factors contribute to this situation, this phenomenon has also technical reasons [62]. It has been highlighted that there is clear misalignment between Moroccan farmers’ preferences and North African breeders’ targets, suggesting that the development of participatory weighted selection (PWS) indices could be implemented to increase the likelihood of acceptance of new releases [61]. Briefly, PWS indices are numerical representation that combine multiple data in a weighted way (i.e., not all traits are given equal importance) taking into account the priorities and preferences of the end-users and are designed to allow local knowledge to be incorporated into the selection process.

Participatory Variety Selection (PVS) is an approach that aims to expedite the adoption of new improved varieties in farmers’ fields by involving farmers in the selection process [63]. PVS complements ongoing, centralized varietal development efforts by offering farmers a broader, often preselected range of germplasm options to evaluate and adopt under their own local conditions. The primary contrast between PVS and PPB revolves around the extent of farmers’ engagement throughout the breeding program. PVS focuses on directly addressing farmers’ requirements, which commonly encompass agronomic and quality traits, with the aim to ensure that the attributes of interest are immediately selected and tailored to their preferences. This approach has proven to be efficient in disseminating new varieties and addressing farmers’ specific requirements that may not be recognized through conventional non-participatory methods of varietal development [64]. Successful implementations of PVS have been reported for maize in Africa and for rice in South Asia [65,66,67]. In barley, PVS has been fruitfully employed for the selection of rainfed barley in Iran [68], malt barley in North–West Ethiopia [69,70], and food barley in Ethiopia [71]. While it should be acknowledged that PVS was not designed as a conservation approach, it is important to recognize that the implementation of PVS can inadvertently impact the conservation of traditional local germplasm. Moreover, in addition to the common restrictions related to resource availability and long-term economic sustainability of participatory and decentralized efforts mentioned before, recognized limits of PVS are issues related to the scale and representativeness (including gender equity considerations) of the stakeholders, and the level of their involvement, which may be a simple visit or visual assessment of experimental stations. Finally, it is accepted that PVS like any participatory selection process may compromise some elements of scientific rigor of plant breeding, such as statistical analysis and replications, posing intrinsic limitations on the evaluation and comparison of the varieties.

### 3.2. Key Policy and Legal Frameworks to Sustain the Conservation and Use of Barley Genetic Resources

In response to the increasing concerns on the loss of diversity in barley and in other crops, international efforts are underway to promote the sustainable use and conservation of barley genetic resources. These frameworks provide guidelines, regulations, and incentives to promote the effective management of plant germplasm. They also facilitate access to genetic resources for research, breeding, and development purposes while ensuring fair benefits for all stakeholders involved. The Convention on Biological Diversity (CBD) established in 1992 and the International Treaty on Plant Genetic Resources for Food and Agriculture (ITPGRFA, also known as the “Plant Treaty”), adopted by FAO in 2001, are arguably the most important international agreements that aim to promote the conservation and sustainable use of biodiversity, including plant genetic resources. They recognize the sovereignty of countries over their genetic resources and are calling the parties to contribute to their conservation and sustainable use and for a fair and equitable sharing of the benefits arising from their use. These two binding agreements call for the development of national strategies and action plans and of national policies and legislations facilitating access to and exchange of genetic resources. An important component is the establishment of legal instruments to regulate access and benefit-sharing in a transparent and fair way. They involve the implementation of material transfer agreements, such as the Standard Material Transfer Agreement (SMTA). This is a private contract developed by the ITPGRFA that defines the terms and conditions for the exchange and use of genetic materials ensuring that countries and organizations that contribute genetic resources are recognized and rewarded for their efforts. Barley and its related *Hordeum* species are part of the Multilateral System of the Plant Treaty, encompassing a total of 26 crops. Moreover, access to germplasm derived from breeding activities, categorized as “Material under Development,” is governed by the SMTA. However, when it comes to collecting additional accessions, two options are available: utilizing the SMTA or adhering to the requirements of the Nagoya Protocol, which involve Prior Informed Consent and Mutually Agreed Terms. The Nagoya Protocol is a supplementary agreement to the CBD for the implementation of measure to access GR and the fair and equitable sharing of benefits arising from their utilization [72]. Just as example, at ICARDA, both genetic resources and material under development are distributed using the SMTA. In the case of the latter, the SMTA also includes provisions for acknowledging the sources of germplasm, sharing information, and sending seed samples to ICARDA.

### 3.3. A Selection of Initiatives and Partnerships for Barley Genetic Resources Management

Collaborative efforts between national and international organizations, research institutions, and farming communities are critical for the sustainable management and use of barley genetic resources. Key milestones in international initiatives for barley genetic resources management and exploitation include (i) the International Barley Genetics Symposium (IBGS) initiated in 1963, which can be considered among the first international platforms for researchers, breeders, and other stakeholders, to share knowledge and advancements in barley genetics and breeding, (ii) the International Barley Genetic Resources Network (IBGRNet), a global network of barley gene banks established in 1986, and (iii) the Barley Genome Project, launched in 2006 and coordinated by the International Barley Genome Sequencing Consortium (IBGS) [73,74].

A prominent on-going initiative is currently the AGENT project (Access to Genetic Resources and Digitization of Plant Genetic Resources). AGENT aims to unleash the untapped potential of the vast biological material stored in gene banks worldwide by leveraging FAIR (Findable, Accessible, Interoperable, and Reusable) international data standards and an open digital infrastructure dedicated to the management of plant genetic resources. The project aims to promote a more systematic effort to exploit Plant Genetic Resources (PGR) and advocate for generating extensive genotypic and phenotypic data for PGR stored in gene banks (GB), thus facilitating their educated selection and utilization in breeding and agriculture. AGENT consortium focuses on the following: (i) the establishment of an actively cooperating gene bank network, (ii) collecting new data and working on agreed standards and protocols for the use of passively stored GB information, (iii) generating new genotypic information for European barley and wheat collections in order to establish a roadmap and pave the way for a complete global wheat and barley biodiversity atlas, (iv) using this extensive genotypic information to evaluate the quality and redundancy of existing PGR collections as a basis for new quality control and management pathways, (v) establishing coordinated PGR training populations for phenotyping of independent collections as a foundation for a pan-European, genome-wide prediction, and (vi) mining new and historic genotypic and phenotypic information to drive the discovery of genes, traits, and knowledge for future missions. Furthermore, at a larger scale, the project aims to increase data density on collections and disseminate the societal impact of PGR to provide the community with a new database and novel data-mining tools to facilitate a well-informed selection of PGR for different purposes (https://www.agent-project.eu/; accessed on 1 July 2023).

An important on-going initiative focused on scaling the impacts of previous efforts in the field of crop wild relatives (CWR) and participatory research is BOLD-DIIVA-PR II project (Dissemination of ICARDA Varieties through Participatory Research). ICARDA breeding programs for durum wheat, barley, and lentil have successfully integrated the use of CWR that possess unique genetic traits that can enhance the adaptability and resilience of cultivated varieties to drought and other environmental stresses prevalent in the regions where ICARDA operates [75]. Building upon the achievements of the DIIVA-PR project, the current project aims to further advance the utilization of CWR-derived lines of durum wheat and barley developed by DIIVA-PR II. The project operates in countries such as Morocco, Tunisia, Ethiopia, Senegal, Nigeria, and Sudan. The objectives of BOLD-DIIVA-PR II are multifold. By involving local farming communities in participatory actions, this project ensures that the developed varieties align with farmers’ preferences and requirements. Additionally, landraces of barley and durum wheat are assessed in the fields for various traits of interest. The best-performing germplasm is then incorporated into breeding programs, considering both performance and farmer preferences. The project strives to generate scientific articles that document the performance of CWR- and landrace-derived elites, facilitate the release of improved varieties in partner countries, and enhance the capacity of local partners in conducting field evaluations. Furthermore, the project promotes open data sharing, ensuring that valuable breeding materials and evaluation data are freely accessible. By engaging stakeholders at various stages, from breeding to evaluation and release, the project strengthens collaboration between research institutions, international organizations, and farming communities, ultimately contributing to the sustainable management and utilization of barley genetic resources.

Finally, although the activity of the following institutions is not limited to barley, it is worth acknowledging the efforts of the European Cooperative Programme for Plant Genetic Resources (ECPGR) Working Group on Barley and the U.S. National Plant Germplasm System (NPGS). The former plays a pivotal role in the conservation and sustainable utilization of barley genetic resources across Europe. This working group brings together experts, scientists, breeders, and stakeholders from various European countries who are dedicated to the conservation, evaluation, and improvement in barley diversity. Within the NPGS, a collaborative effort to preserve the genetic diversity of economically significant plant species, the Plant Genetic Resources Unit (PGRU) and its partners are responsible for collecting, preserving, and distributing barley genetic material worldwide. NPGS collections are managed by the Agricultural Research Service (ARS) of the United States Department of Agriculture (USDA).

## 4. Exploring and Utilizing Barley Genetic Resources at ICARDA: A Historical Appraisal

ICARDA, a member of the CGIAR, was established in 1977 and holds the global mandate for the improvement in barley. This means that this non-profit agricultural research institute is recognized as the leading organization responsible for conducting research, developing strategies, and implementing programs aimed at enhancing the genetic potential and overall performance of barley crops worldwide, in addition to ensuring the conservation of barley genetic resources. Table 1 presents the composition of the barley collection at ICARDA. Besides cultivated and wild barley, the collection comprises the following: *H. murinum* L., adapted to saline environments and serving as genetic resource for studying and improving salt tolerance [76]; *H. bulbosum* Sieber ex Kunth, a perennial species of applied interest as a source of traits to improve biotic stress resistance [77]; *H. marinum* Huds., primarily found in coastal and maritime habitats, of interest to gain insights into the adaptation to saline environments [78]; *H. brevisubulatum* Link and *H. geniculatum* All., two halophytes studied as a potential source of tolerance to drought and other abiotic stress such as alkalinity [79,80]; and *H. turkestanicum* R.E. Regel and *H. hrasdanicum* (part of *H. murinum* subsp. *leporinum* (Link) Arcang.) [81] thought to be adapted to diverse environments, hence holding potential to provide insights into barley responses to abiotic stress [82,83].

The Genetic Resources Unit (GRU) was established in 1983 to safeguard the genetic resources of all the ICARDA mandate crops, through germplasm exploration, gathering, evaluation, and conservation [84]. The GRU also actively works to support ICARDA and NARS breeders through various research activities. They include phenotyping and genotyping resources to identify critical resistance traits that can be used in breeding programs; pre-breeding initiatives to introgress adaptive traits from wild relatives and landraces into elite germplasm of mandated crops with the goal of ensuring yield stability, quality, and nutritional attributes, contributing to the development of new open access tools for electronic data capture, analysis, and decision support. In addition to the core activities of the ICARDA GRU and GB, several collaborative projects have demonstrated the efficiency of using CWR in both pre-breeding and breeding programs for rainfed cereal-based production systems. 

For barley, the routine pre-breeding activities at ICARDA focus on the identification and use of landraces and CWR, such as *H. spontaneum* and *H. bulbosum*, as sources of disease resistance, abiotic stress tolerance, and end-use quality. ICARDA’s work on landraces began in Syria in 1984 [85], at the time host nation of the ICARDA headquarters. The work continued by testing pure lines extracted from landrace populations with the aim of deploying useful traits into adapted genetic backgrounds [32,86]. The objectives of utilizing landrace genetic variability and adaptability were twofold: first, to develop new varieties for Syria, where conventional breeding had encountered challenges, and second, to develop a methodology for landrace utilization adaptable to different crops and regions, particularly for breeders from developing countries with limited resources [32]. The inclusion of CWR became an integral part of ICARDA GRU’s strategy in the 1980s when researchers focused their interest on mining beneficial alleles in untapped germplasm, particularly *H. spontaneum*, due to its crossability with *H. vulgare*. In 2004, ICARDA released six barley cultivars with drought tolerance derived from *H. spontaneum* for use in Syria [87,88]. With the advent of molecular marker technology, crosses between elite cultivars and *H. spontaneum* were also used to dissect complex traits such as drought stress [89]. For example, a study using a subset of the same germplasm tested in multi-environment trials showed that there was variation in grain yield under drought stress among barley genotypes [90], and some of the lines derived from *H. spontaneum* consistently exhibited superior specific adaptation to the range of severe stress conditions employed [90]. Other studies focused on the identification of QTL for agronomic traits in Mediterranean environments [89], dryland characters [91], straw quality [92], powdery mildew [93], and leaf scald [94]. In addition to abiotic stress tolerance, disease resistance plays a crucial role in the development of new breeding lines, particularly for smallholder farmers who often face challenges in accessing and affording fungicides. For instance, a screening of 307 accessions of *H. spontaneum* from different origins, introgression lines derived from crosses between different barley cultivars (Morex, Golden Promise, Vada, and Emir), and lines derived from interspecific crosses with both *H. spontaneum* and *H. bulbosum*, was carried out in two cropping seasons at four experimental stations in Morocco [95]. The germplasm was evaluated for field reactions to powdery mildew, leaf scald, leaf rust, and the net form of net blotch. Specifically, powdery mildew resistance was screened at key locations under natural occurrence, while for the other diseases, plants were tested with mixtures of different isolates collected at different locations in Morocco using spreader rows. Among the materials tested, only three accessions of *H. spontaneum* showed high resistance levels to all four diseases, while 23 other accessions and 16 *H. bulbosum*-derived lines showed resistance to a combination of two to four diseases. Despite the good resistance levels found for individual diseases, the agronomic performance of all lines never surpassed that of the checks used, highlighting the importance of crossing selections from landraces and CWR derivatives with elite adapted materials [95]. 

The GRU is also working on the nutritional content of barley germplasm. For instance, the beta-glucan and microelement content (iron, zinc, selenium) were recently assessed in a panel of cultivated barley and *H. spontaneum* accessions [96]. The results showed that *H. spontaneum* accessions have higher beta-glucan content, while no substantial differences were found in microelements, except for a few *H. spontaneum* lines that showed higher combined contents of iron, zinc, and selenium. These accessions are currently being used in interspecific crosses to develop biofortified barley germplasm and eventually new varieties.

The choice of accessions from ICARDA, as well as from other gene banks, has been typically carried out using random sampling or core collections. The former involves selecting accessions without any specific criteria, while core collections are designed to capture a significant proportion of the genetic diversity within 10% of the total accessions [97]. To further enhance the efficiency and representativeness of core collections, the Generation Challenge Program (GCP), a global research initiative launched in 2004, introduced the concept of a reference set based on molecular markers and genomics technologies. The GCP proposed developing a reference set that would encompass 70% of the genetic diversity found within the core collection but in a more manageable subset, consisting of only 10% of the core collection. Without undermining the vastness and usefulness of the whole genetic resources available in gene banks, statistical and computational techniques to analyze large datasets and identify patterns or relationships between traits and genetic markers are today deemed necessary to construct ‘best bet’ subsets. These are carefully selected subsets of non-overlapping genetic resources that have a high probability of possessing the desired traits with a variability adequate to achieve the best interpretability and exploitability of the feature of interest. 

The Focused Identification of Germplasm Strategy (FIGS) is an approach developed by ICARDA in collaboration with the Vavilov Institute in Russia and GRDC-Australia to efficiently identify and prioritize crop germplasm collections based on specific target environments and traits [98]. It was developed in the late 1990s and has since been widely adopted in plant genetic resources management. FIGS involves a systematic process of collecting and characterizing germplasm resources, particularly landraces and wild relatives, to identify those with desirable traits that are well adapted to specific agroecological conditions. The strategy considers factors such as climate, soil, and farming systems to define target environments, and traits of interest may include tolerance to abiotic stresses (e.g., drought, heat) or biotic stresses (e.g., pests, diseases), as well as specific quality attributes. Technically, FIGS is based on an algorithm that matches plant traits with geographic and agro-climatic information of the places where samples were collected. It creates ‘best-bet’ trait-specific subsets of material by passing accession-level information, especially agro-climatic site information, through a series of filters that increase the chances of finding the adaptive trait of interest. For instance, by utilizing eco-geographic parameters linked to the original collection sites, climate modelling techniques were employed for trait mining among barley accessions for resistance to net blotch (*Pyrenophora teres* Drechs. f. *teres*), illustrating the potential of using freely available databases to enhance the efficiency of field screening trials [99]. Recently the FIGS has been improved by combining it with machine learning methods for a predictive characterization of GB materials for agronomic traits and for grain morphological parameters. The high predictability of models for all traits was later used for predicting the non-evaluated accessions and assigning probabilities for the characterization traits. These results were used as predictive characterization [98]. A further step towards the fine tuning of FIGS approach using machine learning has been recently carried out [100]. The study compared two different subsets of potentially scald-resistant germplasm, one selected with GCP while the second was selected using the FIGS approach. Seedling and adult plant evaluation in multi-environmental trials for scald resistance was performed for both sets, and results showed that the FIGS approach was able to capture higher percentages of resistant accessions compared to the GCP subset. Furthermore, machine learning models tuned on training sets were used to predict scald response on test sets. All models efficiently identified resistant accessions with specificities higher than 0.88 but showed different performances between isolates at the seedling and to field populations at the adult plant stage. 

## 5. Conclusions and Perspectives

The procedures and approaches for the conservation and use of barley genetic resources described here highlight some crucial aspects that need to be addressed, such as the need to align conservation strategies with continuous progress in cultivation practices, particularly in developing countries [101,102]. Moreover, the demand to unlock genetic resources for breeding remains strong due to the ongoing development of improved varieties also suitable for smallholder farmers [101]. These aspects center around identifying emerging trends in the local diffusion of barley varieties, understanding the adoption of new technologies, and recognizing the changing preferences of farmers [32,103].

In barley, the creation and distribution of improved varieties in various areas of the world is still one of the key goals of the breeding efforts of international institutions [104]. While developing countries continue to face well-known specific conditions in agriculture (such as limited resources, vulnerable agro-ecological environments, and unstable socio-economic contexts), new improved varieties of certain crops, including barley, are becoming more widely adopted than in the past [105,106]. Varieties with enhanced traits such as yield, disease resistance, and quality attributes, as well as adaptation to specific agro-ecological conditions, are offering advantages to farmers, leading to higher crop yields and potentially higher income. Correspondingly, traditional farming methods are increasingly integrating modern agricultural techniques systems [107]. This convergence blends knowledge and agro-techniques, resulting in more efficient yet not strictly traditional farming systems [107]. In this globally changing scenario, it is important to consider the potential negative implications of a widespread shift towards improved varieties on the conservation of landraces [103]. To address the dilemma between the loss of barley biodiversity and the promotion of large-scale adoption of new varieties, it is therefore necessary to strengthen the conservation of barley genetic resources using complementary ex situ and in situ approaches. Conservation efforts will continue to be central, but strategies and the support of local farming systems need to be updated to effectively address changing needs [108]. Considering also the threats faced by natural habitats and traditional farming systems due to climate change, urbanization, and land-use change, a shift towards an enhanced ex situ conservation will have to represent a substantial transformation in the conservation strategies of barley [109,110,111]. This could involve the adoption of cutting-edge technologies for seed storage, including automated or robotic high-density storage facilities, the expansion of conserved barley material diversity, and the enhancement of documentation and data management through advanced systems and digital databases, while also minimizing redundancy. Additionally, this endeavor entails the development of more efficient, standardized, and transferable approaches for characterizing and sharing genetic resources among research institutions and breeding programs.

While in situ conservation will always remain crucial for maintaining the dynamic interactions between barley and its natural environment, in the future, it will be necessary to strengthen policies to define and manage controlled environments where barley genetic material can be stored under specific conditions. It is also necessary to further recognize the importance of biodiversity hotspots worldwide. These should be given special attention, included in protected areas, and better managed for the conservation of biodiversity.

Relevant advancements are expected to increase our understanding of the genetic variation of barley germplasm [112]. Briefly, this is being expanded by the continuous emergence of new genomics tools, including advancements in long-read DNA sequencing technologies, bioinformatics analysis, and the digitalization of bioresources [113]. Omics-based approaches on barley genetic resources can help unravel the molecular mechanisms underlying important adaptive traits and provide a deeper understanding of how genetic variation influences the phenotype [112,114]. However, these technologies often require substantial investments in infrastructure, equipment, and expertise, making them less accessible to researchers and breeding programs with limited resources. This may result in disparities in the ability to fully exploit the genetic potential of barley germplasm across different regions or institutions. Staying updated and integrating new techniques into existing workflows requires continuous training and investment in research and development, which may be a logistical and financial burden for some institutions or breeding programs. The challenges posed by highly technological approaches in the characterization of barley germplasm make it difficult to predict how they will be tackled. There are two potential approaches: a more centralized style or an increase in networks. The centralized approach would involve establishing a handful of laboratories with innovative, highly automated, advanced phenotypic and genotypic technologies, which should set and promote coordination and standardization across the globe. On the other hand, expanding networks and ensuring widespread access to cutting-edge technologies should be implemented if the aim is to foster collaboration, inclusivity, and capacity building. It is likely that a combination of both approaches would be the most effective strategy, leveraging the strengths of centralized expertise and distributed knowledge networks. Ultimately, the success will depend not only on willingness to collaborate, but on investment in capacity building to ensure the effective and shared utilization of barley genetic resources. 

To enhance the effectiveness of conservation efforts, it is also crucial to establish a strong connection between the conservation and utilization of barley genetic resources. As an example, an approach to achieve this is by improving the Focused Identification of Germplasm Strategy (FIGS) [101,115]. Furthermore, it is essential to strengthen pre-breeding initiatives by incorporating climate-resilient and nutritionally rich genes from both landraces and wild relatives. By tightly linking conservation with the utilization of genetic resources, we can effectively harness the potential of landraces and wild relatives to develop climate-resilient and nutritionally superior barley lines. 

In conclusion, now more than ever, it is an integrated approach that can ensure that genetic resources are not only preserved but also actively utilized to address the challenges of sustainable agriculture and food security [116]. Therefore, we believe that a complementary genotypic and phenotypic evaluation of gene bank collections will be the next crucial step towards tapping into the vast untapped biodiversity of barley genetic resources. From an applied perspective, this approach will allow for a targeted selection of accessions for pre-breeding purposes from the massive pool of currently available genetic resources. Considering the needs of traditional farming, we believe that it may be necessary to go beyond grain yield improvement as indicator of profitability, lifting the selection focus from allele mining to a more comprehensive genome-wide selection strategy for adaptability. Implementing these proposed activities requires a long-term commitment, and therefore, only sustained funding and collaborative efforts (involving multiple institutions and stakeholders) may embrace this strategy effectively. The commitment to ongoing research, collaboration, and the utilization of advanced breeding techniques will be key in ensuring the sustainable and efficient use of genetic resources for the development of improved barley varieties with enhanced grain yield and other desirable traits.

## Figures and Tables

**Table 1:** Composition of the barley collection at ICARDA.

Taxon	Common Name	Accessions (n)
*Hordeum vulgare* subsp. *vulgare*	Cultivated barley	30,215
*Hordeum vulgare* subsp. *spontaneum*	Wild barley	2005
*Hordeum murinum*	wall barley or mouse barley	284
*Hordeum bulbosum*	bulbous barley	197
*Hordeum marinum*	sea barley	54
*Hordeum brevisubulatum*	short-awned barley	16
*Hordeum turkestanicum*	syn: *H. brevisubulatum* subsp. *turkestanicum* Tzvelev	6
*Hordeum geniculatum*	kneed barley	4
*Hordeum hrasdanicum*	synonym: *H. murinum* subsp. *leporinum*	3
Other		6
Grand total		32,790

## Data Availability

Not applicable.
